# Mosquera’s Food Products, Beef Meal, Beef Cacao

**Published:** 1891-01

**Authors:** 


### MOSQUERA'S FOOD PRODUCTS—BEEF MEAL, BEEF CACAO.

Parke, Davis & Co., whose reputation for orginal work has long been established, announce that after thorough study of the various food products they can now supply preparations which will fulfil all the requirements for therapeutic and dietetic use.

Physicians, in their practice, very frequently meet with cases where nutrition is of more importance than medication; in fact, cases where nutrition is the only agent they can count upon The question of replacing the waste of tissue, where normal nutrition is inefficient, by means of concentrated or predigested foods, is one that always presents many difficulties, there being very few preparations, if any, that meet all the requirements of the medical profession.

Heretofore medical practitioners have had at their disposal a great variety of preparations of meat. These are divisible into four great classes. We have, in the first place, the extracts of meat, prepared after the formula of Liebig; then the so called meat juices: next the ordinary powdered meats; and, finally, the meat peptones.

The ordinary process of preparing meat extracts involves a simple extraction of meat with either warm or cold water, and an evaporation of the resulting solution continued until reduced to a thick liquid or paste. This extract contains the inorganic soluble salts of the meat and some stimuiating organic matter, but none of the nourishing, flesh-forming albuminous substance.

The meat juices are merely cold extractions of the meat, and such products contain some soluble albumen, which coagulates out upon boiling, and naturally, cannot amount to much more than four or five per cent. Tbe meat juices, therefore, possess but little nutritive value.

Powdered meats, as heretofore known, are nothing more nor less than the residue left after extracting all the soluble constituents. Dujardin-Beaumetz and several therapeutists, as a result of a careful line of experiments, concluded that this powder posessed a high nutritive value, and could be employed to advantage in the treatment of certain diseases (consumption and dyspepsia especially). That they are concentrated nutrients is a fact, for beef, in its natural condition, contains 75 per cent. of moisture, all of which is driven off in the preperation of the powder. The fact, however, that these powders are liable to become rancid, or else have been deprived of the inorganic salts, peculiar to meat in its natural state, which salts are quite essential in the digestive process, is an objection to the meat in this form. Moreover, powdered beef requires just as much effort on the part of the stomach to digest it, as does ordinary beef, and for this reason cannot be regarded as proper food for patients suffering with derangement or weakness of the digestive organs.

Another group of meat preparations embraces the meat peptones.

Peptone is the ultimate product of digestion, and the form in which the albuminous or proteid matter is assimilated by the system. These peptones are invariably the product of the artificial digestion of meat by animal pepsin and hydrochloric acid, or, although to a smaller extent, by the digestive ferment of the carica papaya. These are the only preparations really valuable as nutrients. But the physician meets here with another difficulty, in many cases insurmountable; the taste of the peptones is, more or less, bitter and objectionable to the palate, so that patients either absolutely refuse to take them, or take them only with the greatest repugnance. Besides this, their price is comparatively so high that frequently the physician is obliged to abstain from prescribing them.

*All the difficulties heretofore encountered by the medical profession in the use of predigested foods, have been overcome by the new food products of the Mosquera-Julia Food Company.*

Mosquera's Beef Meal contains all the stimulating principles of the extracts of meat, and, in addition, the nutritive principles which the extracts lack; all the albumen of meat juices without their weakness; all the strength of powdered meats without their rancidity and insolubility; all the peptone of the peptonized meats without their bitterness.

The claims we make on behalf of Mosquera's Beef Meal, therefore, cannot be over estimated; they are based on its analysis and properties, and may be condensed as follows:

Mosquera's Beef Meal is a perfectly pure predigested meat, containing all the nutritious constituents of good lean beef, half of which are in soluble form, ready for immediate assimilation, and the other half easily digestible by the gastric and pancreatic juices. Therefore the entire preparation, being practically dry, is composed of nutritive matter, containing about 40 per cent. of soluble peptone and albumose.

It represents, in actual nutritive value, at least six times its weight of good lean beef.

It is perfectly palatable, and will be tolerated with ease by the most delicate stomach.

It admits of being administered in a variety of forms, thus avoiding monotony in the food.

It is the most nutritious as well as the most economical concentrated food.

It must be understood that Mosquera's Beef Meal is not a ready prepared dish, but rather a raw product. It is nothing more than a concentrated beef, converted by artificial digestion into a form which renders it assimilable upon mere contact with the mucous membranes of the alimentary canal.

It, therefore, must be treated by the nurse or cook with the same regard to flavor and taste they would exhibit in the preparation of beef steak Ordinary beef, if simply boiled in water, would neither yield a palatable bouillion nor be eaten itself; salt and other condiments must be added to it. So also, in the use of this beef meal, ingenuity has necessarily to be exercised in its preparation. No matter how palatable or nutritious a food may be, unless presented in a variety of forms it will inevitably become monotonous and even repulsive, this being especially true with patients whose digestive organs are in a weak and debilitated condition. If, therefore, a patient is to take the beef meal for a length of time, it must be administered in a variety of forms to insure the benefit of all its nutritious value.

It may be given in different soups, condimented to suit the taste of the patient, as also mixed with biscuit powder or oatmeal porridge and milk and sugar. Again, it may be mixed with chocolate, which makes a delicious beverage, or given in the form of a sandwich, and finally as a plain beef tea, simply dissolving it in hot water, adding salt.

Mosquera's Beef Cacao consists of equal parts of beef meal, sugar and superior article of Dutch cacao. It does not require cooking, but may be mixed with warm milk exactly like ordinary chocolate, and so completely is the taste of the beef disguised that it cannot be detected. Requiring therefore no previous preparation it is most conveniently administered.

To physicians interested a pamphlet fully discrirtive of the special advantages, uses and methods. of administration of these preparations will be mailed on request, and samples will be sent to physicians who desire to clinically test them in practice,

---

### THE SQUEEZING POINT.

At a recent examination of lady graduates the following question is said to have been given: "If thirty-two is the freezing point what is the squeezing point?" "Two in the shade" was the answer that received the highest marks.—Ex.

